# Preliminary *In Vitro* and *In Vivo* Evaluation of Antidiabetic Activity of *Ducrosia anethifolia* Boiss. and Its Linear Furanocoumarins

**DOI:** 10.1155/2014/480545

**Published:** 2014-03-31

**Authors:** Nagwa M. M. Shalaby, Howaida I. Abd-Alla, Hanan F. Aly, Marzougah A. Albalawy, Kamel H. Shaker, Jalloul Bouajila

**Affiliations:** ^1^Natural Compounds Chemistry Department, National Research Centre, Dokki, Giza 12622, Egypt; ^2^Therapeutic Chemistry Department, National Research Centre, Dokki, Giza 12622, Egypt; ^3^University of Tabuk, Tabuk 71491, Saudi Arabia; ^4^Chemistry Department, Faculty of Science, King Khalid University, Abha 9004, Saudi Arabia; ^5^Faculté de Pharmacie de Toulouse, Laboratoire des IMRCP UMR CNRS 5623, Université de Toulouse, Université Paul-Sabatier, 118 Route de Narbonne, 31062 Toulouse, France

## Abstract

*Aim*. *Ducrosia anethifolia* is used as flavoring additive. There have been little detailed phytochemical reports on this genus and the antidiabetic activity of this plant is not yet evaluated. * Method*. Structure of compounds was deduced by spectroscopic analyses. Preliminary *in vitro* evaluation of the antidiabetic activity of crude extract and its furanocoumarins was carried out (**α**-amylase, **α**-glucosidase, and **β**-galactosidase). The *in vivo* activity was investigated by measuring some oxidative stress markers. Biomarkers of liver injury and kidney were also determined. *Results*. Eight linear furanocoumarins, psoralen, 5-methoxypsoralen, 8-methoxypsoralen, imperatorin, isooxypeucedanin, pabulenol, oxypeucedanin methanolate, oxypeucedanin hydrate, and 3-*O*-glucopyranosyl-**β**-sitosterol, were isolated. All compounds were reported for the first time from the genus *Ducrosia* except pabulenol. The blood glucose level, liver function enzymes, total protein, lipid, and cholesterol levels were significantly normalized by extract treatment. The antioxidant markers, glucolytic, and gluconeogenic enzymes were significantly ameliorated and the elevated level of kidney biomarkers in the diabetic groups was restored. The compounds showed inhibitory activity in a concentration dependant manner. Imperatorin and 5-methoxypsoralen showed the most potent inhibiting power. *Conclusion*. *D. anethifolia* extract showed hypoglycemic, hypolipidemic, and antioxidant effect as well as ameliorating kidney function. This extract and some linear furanocoumarins exhibited carbohydrate metabolizing enzymes inhibitory effect.

## 1. Introduction

Furanocoumarins are well-known natural products that occur in most evolved genera of the Apiaceae family. These compounds were found to have antitumor, phytotoxic, photosensitizing, antibacterial, and high antifungal effects [1, 2].

A large number of studies are in progress to find natural sources, which are effective in reducing the intensity of diabetes [3]. There are some examples of coumarin natural products that exert antidiabetic effect [1, 3]. Osthole, a coumarin derivative, reported as antidiabetic agent, alleviates hyperglycemia in* db*/*db* mice [3]. The effect of the psoralen derivatives from radix* Angelicae dahuricae* on retinoid X receptor-*α* enzymes transcriptional regulation was reported [4] and this in turn plays key roles in various biological processes including diabetes and obesity. Shin et al. [5] considered the furanocoumarin byakangelicin to be an inhibitor of aldose reductase for the treatment of diabetic cataract. Pangelin (pabulenol) was reported as an antihyperglycemic component isolated from the roots of* Paeonia lactiflora* [1].

*Ducrosia anethifolia* Boiss. belongs to the Apiaceae family. This aromatic herb is used as a flavoring additive in foods, beverages, and various desserts and as fragrances in cosmetics [6]. The whole herb especially its aerial parts has been used in Iranian folklore medicine as an analgesic and pain reliever for headache, backache, colic, and colds and claimed to be especially effective against anxiety and insomnia.

*Ducrosia* species were investigated mainly for their oil analysis and their biological activities [6]. There have been little detailed phytochemical reports on this genus. A furanocoumarin pangelin and a monoterpene glucoside 8-debenzoylpaeoniflorin were isolated from the aerial parts of* D. anethifolia* [2]. Herein, we investigated the phytochemical constituents of* D. anethifolia* and the potential antidiabetic activity of its crude extract and its major isolated linear furanocoumarins.

## 2. Materials and Methods

### 2.1. General

Precoated silica gel 60 F_254_ plates (Merck) for thin layer chromatography (TLC) were used. TLC spots were visualized under UV (254 nm) and sprayed with convenient spray reagent. Column chromatography (CC) was carried out using silica gel (Si) 60 mesh of 35–60 and 60–120 (E. Merck, Darmstadt, Germany). Electrothermal digital apparatus and Gallenkamp electrothermal melting point apparatus were used. UV analyses for pure samples were recorded, separately, in methanol solutions on UV spectra and were measured using a Shimadzu UV 240 spectrophotometer (P/N 240–58000). Electrospray ionization mass spectrometry (ESIMS) measurement was run on a double focusing Mat 95 sector field mass spectrometer (Finnigan, Bremen, Germany). Also, ESIMS and high-resolution electrospray ionization mass spectrometry (HRESIMS) were measured with Orbital XL (Thermo Fisher, San Jose, CA, USA) and electron ionization mass spectrometry (EIMS) with a Finnigan MAT 8500 was used. The NMR spectra were recorded at 300 (^1^H) and 75 (^13^C) MHz, on a Varian Mercury 300 (Varian, UK) NMR spectrometer and *δ*-values are reported as ppm relative to TMS in the convenient solvent.

### 2.2. Chemicals

The chemicals were high analytical grade products from Sigma (USA), Merck (Germany), BDH (England), Riedel de Hàen (Germany), and Fluka (Switzerland). The kits used were of Biosystems (Spain), Sigma (USA), and Biodiagnostic (Egypt). Purified carbohydrate metabolizing enzymes, *α*-amylase, *α*-glucosidase, and *β*-galactosidase (EC3.2.1.1, EC3.2.1.20, and EC3.2.1.23, resp., Sigma (USA)) were used.

### 2.3. Solvent Systems and Spray Reagents

S_1_: C_6_H_6_/EtOAc (9.5 : 0.5); S_2_: petroleum ether/MeOH (9.5 : 0.5); S_3_: C_6_H_6_/EtOAc (9 : 2); S_4_: CHCl_3_/MeOH (9 : 3) were used for TLC. R_1_: iodine/potassium iodide (I_2_/KI) and R_2_: sulphuric acid/MeOH (30%) were used as spray reagents.

### 2.4. Phytochemical Study

#### 2.4.1. Plant Material

Aerial parts (leaves and stems) of* Ducrosia anethifolia* Boiss. were collected from Ad-Dalfaàh, Mintaqat Al-Qasim, Saudi Arabia, in April 2010. The plant was identified by Dr. Amal M. Fakhri Abdel Salam, Plant Culture Biology Department, Tabuk University, Tabuk, Saudi Arabia. A voucher specimen is deposited at the herbarium of National Research Centre, Cairo, Egypt.

#### 2.4.2. Extraction and Isolation

The air-dried and powdered aerial parts (leaves and stems) of* D. anethifolia *(2 kg) were defatted with light petroleum (60–80°C). The defatted residue was exhaustively extracted with ethanol (80%). The ethanolic extract was evaporated* in vacuo* at 50°C and yielded a greenish black residue (619.1 g). This residue was desalted by precipitation with excess ethanol followed by drying of the filtrate* in vacuo *to give 231.6 g. An aliquot of the dried filtrate (120 g) was fractionated on a Si column using toluene, ethyl acetate, and gradient increasing polarity with chloroform/methanol. The ethyl acetate fraction (38.6 g) was chromatographed on a Si column using stepwise gradient elution with toluene/EtOAc (100 : 0~0 : 100), leading to desorption of three main collective fractions (Fr. I–III), which were separately collected. Fraction I (0–5% EtOAc, 0.4 g) was chromatographed on Si column and eluted with petroleum ether/EtOAc (9 : 1) to give a compound which was purified by crystallization with methanol to give** 1 **(72 mg). Fraction II (10–15% EtOAc, 0.19 g) was subjected to Si column using* n*-hexane/EtOAc (9 : 1) as eluent to give** 2 **(80 mg). Fraction III (20–25% EtOAc, 4.6 g) was applied to Si column using toluene/EtOAc (8 : 2) as eluent, giving five subfractions (III-1 to III-5). Compounds** 3** (83 mg) and** 4** (45 mg) were obtained by crystallization (methanol) from the subfractions III-1 and III-2, respectively. Subfractions III-3 to III-5 were, separately, subjected to repeated column chromatography on Si with* n*-hexane/ethyl acetate to give three semipure compounds. Each compound was crystallized with methanol to give** 5** (0.66 g),** 6 **(0.39 g), and** 7** (0.47 g), respectively. On the other hand, the fraction eluted by chloroform/methanol (9 : 4) (60.2 g) gave two main subfractions. The first one contained mainly** 8 **and traces of** 9**, while the second one contained** 9 **as the major compound. The first subfraction was then subjected to Si column chromatography using the same eluent and final purification was achieved through crystallization with methanol, ethyl acetate, and two drops of benzene to give** 8 **(2.03 g). Purification of** 9** was achieved through fractionation of the second subfraction on Si column with CH_2_Cl_2_/MeOH, resulting in pure** 9 **(22 mg). The purity of compounds** 1**–**4** was being checked by TLC using solvent system S_1_ for compounds** 1** and** 2**, while S_2_ was used for compounds** 3** and** 4**. S_3_ was used for compounds** 5**–**8** and S_4_ was used for compound** 9**. Spray reagent R_1_ was used for compounds** 1**–**8 **and R_2_ was used for compound** 9**. All compounds were characterized mainly by spectroscopic methods, UV, MS, 1H, and ^13^C NMR, as well as comparison of the melting points with authentic samples or those in the literatures.

#### 2.4.3. Isolated Furanocoumarins

The spectral data of the isolated furanocoumarins were illustrated as follows.

 Compound** 1 **(psoralen): C_11_H_6_O_3_; white crystals; m.p. 168-169°C;* R*_*f*_: 0.78 (S_2_); UV: *λ*_max⁡_ (MeOH): 246, 294, 334 sh.; MS* m/z* (100%): 186 [M^+^, 94.1], 158 [M^+^-CO, 100], 130 [M^+^-2CO, 23.9], 102 [M^+^-3CO, 49.5]; ^1^H NMR (CDCl_3_, 300 MHz): *δ* ppm 7.78 (1 H, d, *J* = 9.5, H-4), 7.68 (1 H, d, *J* = 2.4, H-2′), 7.66 (1 H, s, H-5), 7.43 (1 H, s, H-8), 6.82 (1 H, d, *J* = 2.4, H-3′), 6.35 (1 H, d, *J* = 9.5, H-3); ^13^C NMR (CDCl_3_, 75 MHz): *δ* ppm 160.9 (C-2), 156.3 (C-7), 151.9 (C-9), 146.8 (C-2′), 144.0 (C-4), 124.8 (C-6), 119.8 (C-5), 115.3 (C-10), 114.5 (C-3), 106.3 (C-3′), 99.7 (C-8).
 Compound** 2 **(5-methoxypsoralen): C_12_H_8_O_4_; whitish yellow needle crystals; m.p. 189-190°C;* R*_*f*_: 0.73 (S_2_); UV: *λ*_max⁡_ (MeOH): 230, 246, 266, 309; MS* m/z* (100%): 216 [M^+^, 100], 201 [M^+^-CH_3_, 35.5], 188 [M^+^-CO, 25.1], 173 [M^+^-CO-CH_3_, 61.6], 145 [M^+^-CH_3_-2CO, 32.2], 89 [C_7_H_5_^+^, 19.1]; ^1^H NMR (CDCl_3_, 300 MHz): *δ* ppm 8.16 (1 H, d, *J* = 10, H-4), 7.60 (1 H, d, *J* = 2.4, H-2′), 7.15 (1 H, s, H-8), 7.03 (1 H, d, *J* = 2.4, H-3′), 6.28 (1 H, d, *J* = 10, H-3), 4.28 (3 H, s, OCH_3_-5); ^13^C NMR (CDCl_3_, 75 MHz): *δ* ppm 161.2 (C-2), 158.4 (C-7), 152.7 (C-9), 149.6 (C-5), 144.8 (C-2′), 139.2 (C-4), 112.7 (C-6), 112.6 (C-3), 106.5 (C-10), 105.0 (C-3′), 93.9 (C-8), 60.1 (OCH_3_-5).
 Compound** 3 **(8-methoxypsoralen): C_12_H_8_O_4_; white crystals; m.p. 146-147°C;* R*_*f*_: 0.63 (S_2_); UV *λ*_max⁡_ (MeOH): 247, 299 nm; MS* m/z *(100%): 216 [M^+^, 100], 201 [M^+^-CH_3_, 4.1], 188 [M^+^-CO, 35.9], 173 [M^+^-CO-CH_3_, 59.3], 158 [M^+^-CO-OCH_3_/+H, 17.3], 145 [M^+^-CH_3_-2CO, 18.9], 89 [C_7_H_5_^+^, 14.4]; ^1^H NMR (CDCl_3_, 300 MHz): *δ* ppm 7.76 (1 H, d, *J* = 9.6, H-4), 7.69 (1 H, d, *J* = 2.1, H-2′), 7.34 (1 H, s, H-5), 6.82 (1 H, d, *J* = 2.1, H-3′), 6.36 (1 H, d, *J* = 9.6, H-3), 4.29 (3 H, s, OCH_3_-8); ^13^C NMR (CDCl_3_, 75 MHz): *δ* ppm 160.4 (C-2), 147.6 (C-7), 146.6 (C-2′), 144.3 (C-4), 143.0 (C-9), 106.7 (C-3′′), 132.8 (C-8), 126.1 (C-6), 116.4 (C-10), 114.7 (C-3), 112.9 (C-5), 106.7 (C-3′), 61.3 (OCH_3_-8).
 Compound** 4 **(imperatorin): C_16_H_14_O_4_; whitish yellow needle crystals; m.p. 102–104°C,* R*_*f*_: 0.53 (S_2_); UV *λ*_max⁡_ (MeOH): 247, 264, 301 sh; MS* m/z* (100%): 271 [M^+^+H, 30.3], 270 [M^+^, 2.2], 202 [M^+^-C_5_H_8_^+^, 100], 174 [202-CO, 31.0], 145 [202-CO-CHO, 14.4], 89 [C_7_H_5_^+^, 24.3]; ^1^H NMR (CDCl_3_, 300 MHz): *δ* ppm 7.75 (1 H, d,* J *= 9.6 Hz, H-4), 7.68 (1 H, d, *J* = 2.1 Hz, H-2′), 7.35 (1 H, s, H-5), 6.80 (1 H, d, *J* = 2.1 Hz, H-3′), 6.34 (1 H, d, *J* = 9.6 Hz, H-3), 5.60 (1 H, m, H-2′′), 5.00 (1 H, d, *J* = 7.2 Hz, H-1′′), 1.73 and 1.71 (6 H, s, 2CH_3_-3′′); ^13^C NMR (CDCl_3_, 75 MHz): *δ* ppm 160.4 (C-2), 148.6 (C-7), 146.5 (C-2′), 144.3 (C-4), 143.8 (C-9), 139.6 (C-3′′), 131.6 (C-8), 125.8 (C-6), 119.7 (C-2′′), 116.4 (C-10), 114.6 (C-3), 113.1 (C-5), 70.1 (C-1′′), 25.7 and 18.1 (2CH_3_-3′′).
 Compound** 5 **(isooxypeucedanin): C_16_H_14_O_5_; white powder; m.p. 149°C;* R*_*f*_: 0.60 (S_3_); UV *λ*_max⁡_ (MeOH): 252.5, 269 sh, 278, 329 nm; MS* m/z *(100%): 286 [M^+^, 100], 287 [M^+^+H, 17.2], 215 [M^+^-C_4_H_7_O^+^, 34.2], 202 [M^+^-C_5_H_10_O^+^, 25.0], 187 [215-CO, 37.2], 174 [202-CO, 12.0], 145 [202-CO-CHO, 16.0], 71 [C_4_H_7_O^+^, 40.0], 43 [C_3_H_7_^+^, 84.1],; ^1^H NMR (CD_3_OD-d_6_ 300 MHz): *δ* ppm 8.29 (1 H, d, *J* = 9.8 Hz, H-4), 7.58 (1 H, d, *J* = 2.4 Hz, H-2′), 7.15 (1 H, s, H-8), 6.82 (1 H, d, *J* = 2.4 Hz, H-3′), 6.29 (1 H, d, *J* = 9.8 Hz, H-3), 5.06 (2 H, s, H-1′′), 2.84 (1 H, sept., H-3′′), 1.16 (6 H, d, *J* = 6.9 Hz, 2CH_3_-4′′/5′′); ^13^C NMR (CD_3_OD-d_6_, 75 MHz): *δ* ppm 208.8 (C-2′′), 160.9 (C-2), 158.0 (C-7), 152.6 (C-9), 147.9 (C-5), 145.5 (C-2′), 139.2 (C-4), 113.6 (C-6), 113.2 (C-3), 107.4 (C-10), 104.1 (C-3′), 95.0 (C-8), 75.0 (C-1′′), 37.4 (C-3′′), 17.9 (2CH_3_-4′′/5′′).
 Compound** 6 **(pabulenol): C_16_H_14_O_5_; white powder; m.p. 128°C;* R*_*f*_: 0.55 (S_3_); UV *λ*_max⁡_ (MeOH): 247, 331 nm; MS* m/z *(100%): 287 [M^+^+H, 4.8], 286 [M^+^, 24.8], 215 [M^+^-C_4_H_7_O^+^, 3.6], 202 [M^+^-C_5_H_10_O^+^, 100], 174 [202-CO, 38.1], 145 [202-CO-CHO, 4.7]; ^1^H NMR (CD_3_OD-d_6_ 300 MHz): *δ* ppm 8.15 (1 H, d, *J* = 9.8 Hz, H-4), 7.57 (1 H, d, *J* = 2.4 Hz, H-2′), 7.10 (1 H, s, H-8), 6.94 (1 H, d, *J* = 2.4 Hz, H-3′), 6.23 (1 H, d, *J* = 9.8 Hz, H-3), 5.17 (1 H, d, *J* = 1 Hz, H_a_-4′′), 5.04 (1 H, d, *J* = 1 Hz, H_b_-4′′), 4.40 (2 H, m, H-1′′), 1.81 (1 H, s, H-5′′); ^13^C NMR (CD_3_OD-d_6_, 75 MHz): *δ* ppm 161.2 (C-2), 158.0 (C-7), 152.5 (C-9), 148.5 (C-5), 145.1 (C-2′), 143.3 (C-3′′), 139.2 (C-4), 114.0 (C-6), 113.3 (C-4′′), 112.8 (C-3), 107.2 (C-10), 104.7 (C-3′), 94.6 (C-8), 75.5 (C-1′′), 74.1 (C-2′′), 18.7 (C-5′′).
 Compound** 7 **(oxypeucedanin methanolate): C_17_H_17_O_6_; white powder; m.p. 126°C;* R*_*f*_: 0.49 (S_3_); UV *λ*_max⁡_ (MeOH): 254, 268, 310 nm; MS* m/z *(100%): 318 [M^+^, 40.0], 319 [M^+^+H, 6.2], 215 [M^+^-C_5_H_10_O_2_^+^, 2.6], 202 [M^+^-C_6_H_12_O_2_^+^, 100], 174 [202-CO, 21.0], 145 [202-CO-CHO, 6.8], 73 [C_4_H_9_O^+^, 95.0]; ^1^H NMR (CD_3_OD-d_6_, 300 MHz): *δ* ppm 8.18 (1 H, d, *J* = 9.8 Hz, H-4), 7.55 (1 H, d, *J* = 2.4 Hz, H-2′), 7.08 (1 H, s, H-8), 6.97 (1 H, d, *J* = 2.4 Hz, H-3′), 6.22 (1 H, d, *J* = 9.8 Hz, H-3), 4.55 (H, dd, *J* = 3.0, 9.9 Hz, H_a_-1′′), 4.35 (1 H, dd, *J* = 3.0, 9.9 Hz, H_b_-1′′), 3.91 (1 H, m, H-2′′), 3.24 (3 H, s, OCH_3_), 1.24 (1 H, s, H-5′′), 1.21 (1 H, s, H-4′′); ^13^C NMR (CD_3_OD-d_6_, 75 MHz): *δ* ppm 161.2 (C-2), 158.1 (C-7), 152.5 (C-9), 148.8 (C-5), 145.0 (C-2′), 139.2 (C-4), 114.0 (C-6), 112.7 (C-3), 107.2 (C-10), 104.9 (C-3′), 94.4 (C-8), 76.1 (C-2′′), 75.9 (C-3′′), 74.2 (C-1′′), 49.2 (C-OCH_3_-3′′), 20.8 (CH_3_-4′′), 20.6 (CH_3_-5′′).
 Compound** 8 **(oxypeucedanin hydrate): C_16_H_16_O_6_; white shiny crystal; m.p. 128°C;* R*_*f*_: 0.44 (S_3_); UV *λ*_max⁡_ (MeOH): 253, 268, 311 nm; MS* m/z *(100%): 304 [M^+^, 36.2], 305 [M^+^+H, 7.4], 289 [M^+^-CH_3_, 4.2], 286 [M^+^-H_2_O, 8.5], 202 [M^+^-C_5_H_12_O_2_^+^, 100], 174 [202-CO, 37.0], 145 [202-CO-CHO, 10.4], 118 [C_8_H_6_O^+^, 7.6], 89 [C_7_H_5_^+^, 9.1], 59 [C_3_H_7_O^+^, 42.0]; ^1^H NMR (CD_3_OD-d_6_ 300 MHz): *δ* ppm 8.25 (1 H, d, *J* = 9.7 Hz, H-4), 7.69 (1 H, d, *J* = 1.8 Hz, H-2′), 6.95 (1 H, s, H-8), 7.10 (1 H, d, H-3′), 6.16 (1 H, d, *J* = 9.7 Hz, H-3), 4.74 and 4.35 (2 H, m, H-1′′), 3.84 (1 H, d, *J* = 8.2 Hz, H-2′′), 1.34 (3 H, s, CH_3_-5′′), 1.29 (3 H, s, CH_3_-4′′); ^13^C NMR (CD_3_OD-d_6_, 75 MHz): *δ* ppm 164.0 (C-2), 160.3 (C-7), 154.3 (C-9), 151.2 (C-5), 147.5 (C-2′), 142.3 (C-4), 115.6 (C-6), 113.4 (C-3), 108.7 (C-10), 107.0 (C-3′), 95.1 (C-8), 78.8 (C-2′′), 76.3 (C-1′′), 73.5 (C-3′′), 28.0 (CH_3_-4′′), 25.8 (CH_3_-5′′).
 Compound** 9 **(3-*O*-glucopyranosyl-*β*-sitosterol): C_35_H_60_O_6_; white powder;* R*_*f*_: 0.62 (S_4_); m.p. 280-281°C; HRESI-MS* m/z* (100%): 1150.6 [2M-H]^−^, 575 [M-H]^−^; ^1^H NMR (DMSO-d_6_, 300 MHz): *δ* ppm 5.32 (1 H, brs, H-6), 4.20 (1 H, d, *J* = 7.8 Hz, H-1′), 3.61 (1 H, m, H-3), 3.40–2.90 (1 H, d, H-2′); ^13^C NMR (DMSO-d_6_, 75 MHz): *δ* ppm 140.4 (C-5), 121.1 (C-6), 100.8 (C-1′), 77.0 (C-3′/5′), 76.7 (C-3), 73.4 (C-2′), 70.0 (C-4′), 61.0 (C-6′), 56.2 (C-14), 55.0 (C-17), 49.6 (C-9), 45.1 (C-24), 41.8 (C-13), 39.7 (C-12), 38.3 (C-4), 36.8 (C-1), 36.2 (C-10), 35.8 (C-20), 33.8 (C-22), 31.4 (C-7/8), 29.5 (C-25), 29.2 (C-2), 28.7 (C-16), 26.1 (C-23), 24.8 (C-15), 22.6 (C-28), 20.9 (C-11), 19.6 (C-26), 18.9 (C-27), 18.6 (C-21), 19.0 (C-19), 11.7 (C-29), 11.6 (C-18).

### 2.5. *In Vitro* Antidiabetic Activity

#### 2.5.1. Carbohydrates Metabolizing Enzymes Inhibition Assay

(*1*)* Determination of *α*-Amylase Inhibitory Activity.* The inhibitory activity of *α*-amylase was determined according to the method described by Ali et al. [7]. The *α*-amylase activity was determined by measuring the absorbance at 540 nm. The *α*-amylase inhibitory activity (%) was defined as the percent decrease in the maltose production rate over the control. Acarbose was used as positive control. The *α*-amylase inhibition was expressed as a percentage of inhibition and calculated from the equation *A*_540_  control − *A*_540_  sample/*A*_540_  control × 100, where *A* is the absorbance.

(*2*)* Determination of *β*-Galactosidase Inhibitory Activity*. The inhibitory activity of *β*-galactosidase was measured by the method of Sanchez and Hardisson [8] and the resulting color of* O*-nitrophenolate ions was measured at 420 nm. A standard curve was performed using different concentrations of* O*-nitrophenol.

(*3*)* Determination of *α*-Glucosidase Inhibitory Activity*. The *α*-glucosidase inhibitory activity was determined according to the method of Kim et al. [9] and the reducing activity was estimated by measuring release of* p-*nitrophenol from* p*-nitrophenyl *α*-D-glucopyranoside at 405 nm. A standard curve was carried out using different concentrations of* p*-nitrophenol.

### 2.6. *In Vivo* Antidiabetic Activity

#### 2.6.1. Animals

Male Wister albino rats of twenty-week age (250 ± 50 g) were selected for this study. They were obtained from the Animal House, National Research Centre, Egypt. All animals were housed in a temperature- (26–29°C) and humidity- (50–60%) controlled environment, in steel mesh cages of ten rats, each on wood-chip bedding, with a fixed light/dark cycle (12 hours), for one week as an acclimatization period with free access of water and food* ad libitum*. Animals were treated daily with total ethanolic extract of* D. anethifolia* and antidiabetic drug for 45 days. Anesthetic procedures and handling with animals complied with the ethical guidelines of Medical Ethical Committee of National Research Centre in Egypt, providing that the animals did not suffer at any stage of the experiment.

#### 2.6.2. Experimental Design

Fifty male albino rats were selected for this study and divided into five groups (ten rats each) as follows: group 1: normal healthy control rats, group 2: normal rats orally treated with 500 mg/kg body weight (BW) total ethanolic extract of* D. anethifolia* for 45 days according to LD_50_ which revealed that the extract is safe till 1.5 g/kg BW, and group 3: diabetic group; where diabetes was induced by streptozocin (STZ). Each rat was injected intraperitoneally with a single dose of STZ (60 mg/kg BW) dissolved in 0.01 M citrate buffer immediately before use [10].

After injection, animals had free access for food and water and given 5% glucose solution to drink overnight to encounter hypoglycemic shock. Animals were checked daily for the presence of glycosuria. Animals were considered to be diabetic if glycosuria was present for 3 consecutive days. After 3 days of STZ injection fasting blood samples were obtained and fasting blood sugar was determined (>300 mg/dL). Hyperglycemic rats were used for the experiment and classified as follows: group 4: diabetic animal oral administered 500 mg/kg BW of* D. anethifolia* total extractfor 45 days and group 5: diabetic rats orally administered the antidiabetic reference drug glibenclamide at a dose of 5 mg/kg BW d for 45 days [10].

#### 2.6.3. Sample Preparations

After 45 days of treatments, rats were fasted overnight (12–14 hours), anesthetized by diethyl ether and blood collected by puncture of the sublingual vein in clean and dry test tube, left 10 minutes to, and centrifuged at 3000 rpm for serum separation. The separated serum was used for biochemical analysis of liver function enzymes, blood glucose level, lipid profile, and total protein content.

#### 2.6.4. Preparation of Tissue Homogenate

After blood collection, rats of each group were sacrificed; the livers were removed immediately, weighed and homogenized in 5–10 volumes bidistilled water by a ratio of 1 : 10 w/v using electrical homogenizer, and centrifuged at 4000 rpm for 15 min. The supernatants were collected and placed in Eppendorf tubes, stored at −20°C, and used for determination of oxidative stress markers (nitric oxide (NO) and malondialdehyde (MDA)), antioxidant (reduced glutathione (GSH) and superoxide dismutase (SOD)), and carbohydrate metabolizing enzymes (hexokinase (HK), pyruvate kinase (PK), and phosphoenolpyruvate carboxy kinase (PEPCK)). The homogenization was carried out as described by Newsholme [11].

#### 2.6.5. Blood Biochemical Analyses

Glucose was determined in serum by colorimetric assay method kits (Biodiagnostic Chemical Company, Cairo, Egypt). Alkaline phosphatase (ALP) enzyme activity was measured by the method of Belfield and Goldberg [12]. The liberated phenol is measured colorimetrically in the presence of 4-aminophenazone and potassium ferricyanide at wavelength of 510 nm. The enzyme activity is expressed in**μ**mol/mL.

#### 2.6.6. The Liver Injury Biomarkers

Aspartate and alanine aminotransferases (AST and ALT) enzyme activities were measured by the method of Javier Gella et al. [13]. Colorimetric determination of AST activity is catalysed by measuring the keto acid oxaloacetate which is formed in its derivative form 2,4-dinitrophenylhydrazone at wavelength of 505 nm, while in ALT determination the keto acid pyruvate formed is measured in its derivative form 2,4 dinitrophenylhydrazone. The enzyme activities are expressed in**μ**mol/mL. Total protein was assayed in serum according to Bradford [14]. Total protein content in the samples reacted with Bradford reagent (coomassie brilliant blue G-250, ethanol orthophosphoric acid) to give a blue colour complex, which is measured colorimetrically at 595 nm and expressed in mg/mL.

#### 2.6.7. Tissue Biochemical Analyses

*Carbohydrates Metabolizing Enzymes*. Hexokinase enzyme assay in liver tissue homogenate was assayed in liver tissue homogenate according to Abrahao-Neto et al. [15]. An aliquot of liver tissue (0.5 g) was homogenized in the extraction medium which consists of 50 mM triethanolamine, 1 mM EDTA, 2 mM MgCl_2,_ and 30 Mm mercaptoethanol (pH 7.5) to yield 10% homogenate and the supernatant was used for enzyme assays. Glucose 6-phosphate formed by the hexokinase reaction is measured by adding glucose-6-phosphate dehydrogenase and nucleoside derived amino acids NADP following NADPH formation. This method minimizes inhibition due to glucose 6-phosphate by oxidizing it to 6-phosphogluconic acid. The rate of change in optical density is measured at 340 nm. The change in optical density should be between 0.005 and 0.020/minute and measured from the second to the tenth minute after adding adenosine triphosphate (ATP). Pyruvate kinase (PK) and phosphoenolpyruvate carboxy kinase (PEPCK) enzyme activities were determined in liver tissue homogenate using the previous method [11] and the liver tissue was homogenized in 5 mL Tris-HCl buffer pH 7.6. The PK assay is based on the coupled enzymatic conversion of phosphoenolpyruvate (PEP) to pyruvate with the reduction of pyruvate to lactate in the presence of lactate dehydrogenase (LDH) and NADH. The decrease in absorbance was read at 340 nm. PEPCK enzyme activity was measured through the oxaloacetate formation from PEP, with NaHCO_3_ serving as the source of CO_2_ and was determined by measuring the oxidation of NADH in the presence of malate dehydrogenase (MDH) at 340 nm. Lactate dehydrogenase (LDH) enzyme activity was determined in liver tissue homogenate according to the method of Bergmeyer et al. [16]. LDH catalyzes the oxidation of lactate to pyruvate with concomitant reduction of NAD to NADH. The equilibrium of the reaction favors the pyruvate to lactate direction. If the reduction of NAD is coupled with the reduction of tetrazolium salt as 2-*p*-iodophenyl-3-*p*-nitrophenyl-5-phenyl tetrazolium chloride (INT) with phenazine methosulfate (PMS) serving as an intermediate election carrier, the result is the formation of formazan of INT. The developed colour was measured at 503 nm. All enzyme activities are expressed in**μ**mol/mg protein/min.

*Oxidative Stress Markers and Antioxidant Enzymes.* Lipid peroxide (LPO)/malondialdehyde (MDA) [17] and nitric oxide (NO) [18] were determined in the tissue liver homogenate. Thiobarbituric acid (TBA) reacts with MDA in acidic medium at temperature 95°C for 30 minutes to form thiobarbituric acid reactive product; the absorbance of the resultant pink product can be measured at 534 nm. MDA is expressed in**μ**mol/min/g tissue. Nitric oxide (NO) in acid medium and in the presence of nitrite formed nitrous acid diazotize with sulphanilamide and the product is coupled with N-(1-naphthyl) ethylenediamine. The resulting azo-dye has a bright reddish-purple colour which can be measured at 540 nm. NO is expressed in**μ**g/g tissue.

Reduced glutathione (GSH) was assayed according to Moron et al. [19]. The method is based on the reduction of 5,5′ dithiobis (2-nitrobenzoic acid) (DTNB) with glutathione to produce a yellow compound. The reduced chromogen is directly proportional to GSH concentration and its absorbance can be measured at 405 nm. GSH is expressed as *μ*g glutathione/mg protein. Superoxidedismutase (SOD) was assayed according to Paoletti et al. [20] in the tissue liver homogenate. SOD determination is based on the oxidation of nicotinamide adenine dinucleotide reduced disodium salt (NADH) mediated by superoxide radical through a free radical chain of reactions involving thiol oxidation and univalent O_2_ reduction. The increase in absorbance with time was read at 560 nm. SOD is expressed in *μ*mol/mg protein/min.

*Lipid Profile and Kidney Markers*. Profiles of the following serum lipids were established: total cholesterol (TC), triglycerides (TG), low-density lipoprotein cholesterol (LDL-c), high-density lipoprotein cholesterol (HDL-c), very-low-density lipoprotein cholesterol (VLDL-c), and total lipid using diagnostic kits (Biodiagnostic Chemical Company, Cairo, Egypt). Total urea and creatinine were carried out using diagnostic kits (Biodiagnostic Chemical Company, Cairo, Egypt).

*Statistical Analyses*. Data were analyzed by comparing values for different treatment groups with the values for individual controls. Results are expressed as mean ± SD. The significant differences among values were analyzed using analysis of variance (one-way Anova) coupled with CoStat Computer Program. Unshared letters indicate significant correlation at *P* < 0.05.

## 3. Results

### 3.1. Phytochemical Investigation

**Figure 1:**
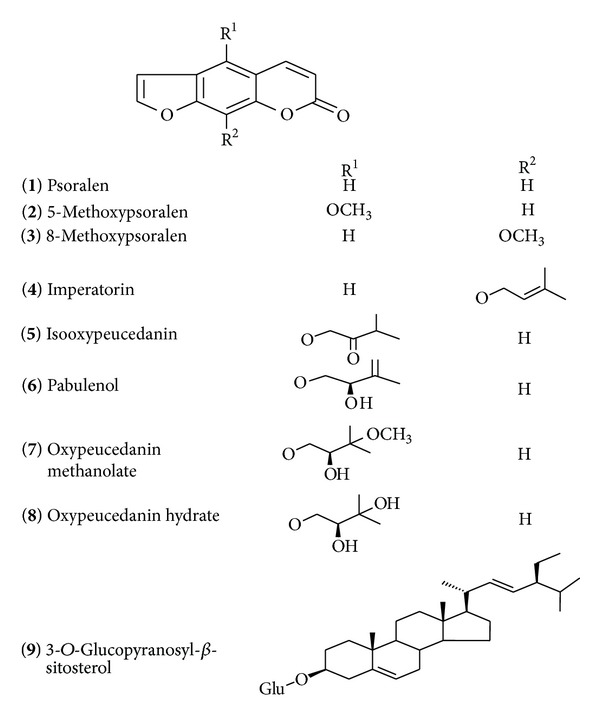
Structures of the isolated compounds from* D. anethifolia.*

The 80% ethanol extract from the aerial parts of* D. anethifolia *was fractionated on a Si column, followed by consecutive purification steps on Si columns to yield eight linear furanocoumarins (**1**–**8**) and one sterol glycoside** 9** (Figure 1).

The isolated compounds were identified as psoralen (**1**), 5-methoxypsoralen (**2**), 8-methoxypsoralen (**3**), imperatorin (**4**), isooxypeucedanin (**5**), pabulenol (**6**), oxypeucedanin methanolate (**7**), oxypeucedanin hydrate (**8**), and 3-O-glucopyranosyl-*β*-sitosterol (**9**).

### 3.2. *In Vitro* and* In Vivo* Antidiabetic Activity

All the tested compounds showed appreciable carbohydrate inhibitory activities. The crude extract showed the highest inhibitory activity of carbohydrate metabolizing enzymes followed by imperatorin and 5-methoxypsoralen. They recorded biologically active inhibition of *α*-amylase, *α*-glucosidase, and *β*-galactosidase enzyme activities at concentrations 10–1000 *μ*g/mL. However, psoralen, oxypeucedanin hydrate, and isooxypeucedanin showed more or less similar moderate percentage of carbohydrate enzyme inhibitory activities. A concentration-response relationship is found in the carbohydrate metabolizing enzyme inhibitory activities. The activity increased in response to the increase in concentration of the tested compound for each individual one (Table 1). From the manipulated results, we can deduce a significant increase in inhibitory power with the increase in concentrations of the compounds (linear relationship) and at low doses the inhibitory activity shows insignificant change.

The present results demonstrate the antidiabetic effect of* D. anethifolia* total extract treatment in comparison to the current available antidiabetic glibenclamide reference drug in diabetic model.  Table 2 manipulated liver function enzyme activities, blood glucose level, and total protein content in normal, STZ, and treated groups. An insignificant change was observed in normal rats treated with* D. anethifolia* total extract as compared to the normal untreated rats (Table 2).

In STZ group, a significant increase was observed in blood glucose level with percentage increase reaching 227.26% and in liver function enzyme activities, AST, ALT, and ALP, with percentages of increase +219.61, +265.40, and +215.03%, respectively. However, a significant decrease was observed in total protein content amounting to −44.25% as compared to the normal control. Significant normalization was noticed in blood glucose level, liver enzymes, AST, ALT, and ALP, and total protein content in diabetic group treated with* D. anethifolia* total extract with percentages of amelioration amounting to 178.78, 194.11, 235.80, 170.52, and 29.98%, respectively, with simultaneous results for glibenclamide-treated diabetic rats (191.42, 207.84, 241.97, 198.55, and 35.35%, resp.) (Table 2).

The data obtained in Table 3 showed insignificant change in lipid profile, total cholesterol, triglycerides, HDL-c, LDL-c, and total lipids in normal control treated rats with* D. anethifolia* total extract. On the other hand, diabetic rats showed significant increase in lipid profile, total cholesterol, triglycerides, LDL-cholesterol, and total lipids with percentages of increase amounting to 222.90, 171.05, 658.42, and 95.54%, respectively, while a significant decrease in HDL-cholesterol was observed (−69.71%), as compared to the normal control group. Treatments with* D. anethifolia* ethanolic extract significantly reversed these elevations and controlled the reduced LDH-c level with percentages of improvement 147.17, 111.65, 30.01, 465.49, and 58.20%, respectively, for total cholesterol, triglycerides, HDL-c, LDL-c, and total lipid. In addition, standard antidiabetic reference glibenclamide drug declared more or less similar results as illustrated in Table 3.

Considering antioxidant markers, an insignificant change was observed in all antioxidant markers after treatment of normal control rats with* D. anethifolia* total extract (Table 4). A significant increase was noticed in NO and MDA after STZ injection with percent of elevation amounting to +79.00 and +569.77%, respectively. A significant reduction was recorded for GSH and SOD with percentages amounting to −70.29 and −83.09%, respectively. It has been easily noticed that significant amelioration in NO, MDA, GSH, and SOD levels after treatment of diabetic rats with* D. anethifolia* total extract was recorded with percentages of amelioration amounting to 62.13, 590.79, 42.35, and 43.83%, respectively, as compared to the antidiabetic standard drug which recorded significant improvement amounting to 62.00, 545.12, 48.82, and 53.95%, respectively, for NO, MDA, GSH, and SOD (Table 4).

With respect to glucolytic and gluconeogenic enzymes, an insignificant change was observed in all enzyme levels as a result of treatment of normal control rats with* D. anethifolia* extract. However, in diabetic rats, significant inhibition in HK, PK, and LDH enzyme activities (with percentages of inhibition of 66.52, 62.62, and 71.27%, resp., accompanied with a significant increase in PEPCK (65.88%)) was recorded in STZ-induced diabetic rats (Table 5). A significant enhancement was noticed in all studied enzymes in treated-diabetic groups either with the total extract of* D. anethifolia* total extract (20.92, 38.45, 29.26 and 41.76%, resp., for HK, PK, LDH, and PEPCK) or with antidiabetic standard drug (39.81, 42.74, 33.07, 50.29%, resp.), as compared to the normal control.

Regarding total urea and creatinine levels, an insignificant change of total urea and creatinine levels in normal treated rats with* D. anethifolia* total extract as compared to normal control group (Table 6) was observed. Diabetic rats showed significant elevation in total urea and creatinine levels with percentages of increase amounting to 147.00 and 364.28%, respectively, as compared to normal control group. Treatment of STZ diabetic rats with crude extract of* D. anethifolia* total extract restored the elevated levels as compared to normal control with percentage of improvement reaching 118.19 and 334.52% for total urea and creatinine, respectively (Table 6). Nearly similar results were obtained for the antidiabetic reference drug (154.05 and 369.05%, resp., for total urea and creatinine).

## 4. Discussion

In this study,* D. anethifolia *was investigated for its phytoconstituents and demonstrated that the crude extract has an antidiabetic effect.

### 4.1. Structural Elucidation of Isolated Compounds

The structures of isolateswere identified on the basis of interpretation of their chemical physicochemical analyses (UV, MS, 1D, and 2D NMR), compariing with the corresponding published data in the literature [21, 22]. Based on the chromatographic properties and UV spectral data the coumarins were identified as psoralen (**1**) [21], 5-methoxypsoralen (**2**) [21], 8-methoxypsoralen (**3**) [21], imperatorin (**4**) [22], isooxypeucedanin (**5**) [22], pabulenol (**6**) [23], oxypeucedanin methanolate (**7**) [22], and oxypeucedanin hydrate (**8**) [23]. The sterol glycoside was 3-*O*-glucopyranosyl-*β*-sitosterol (**9**) [24].

Compounds** 1**–**8** exhibited UV absorption characteristic of linear furanocoumarins [22]. UV spectral data of** 1**–**8** showed absorption maxima at range of 247–269 and 294–334 nm which were characteristic of linear type of furanocoumarins [2]. The mass spectrum supports the UV data. The presence of diagnostic fragment ions at* m/z* 201, 202, 174, and 145 in the mass spectra suggests a furanocoumarin moiety [22]. The ^1^H NMR spectral data for compounds** 2**,** 5**–**8** were characteristic of a 5-*O*-substituted furanocoumarin and compounds** 3** and** 4** were of 8-substituted one. They all confirmed the typical pattern of a linear furanocoumarins [2]. The resonance signals due to H-3 and H-4 appeared as doublets with* J* = 9.6–9.8 Hz; the signal due to H-3 was observed at *δ* 6.16–6.36. The chemical shift of H-4 distinguished the C-8 and C-5 substituted furanocoumarins. In the spectra of compounds** 2**,** 5**–**8**, the resonance signal due to H-4 appeared at *δ* 8.15–8.29, indicating that they were substituted at C-5 [23]; the signal due to H-8 in these compounds appeared at *δ* 6.95–7.15. On the other hand, ^1^H NMR spectra of compounds** 3 **and** 4** contained resonance signals due to H-4, relatively up field at *δ* 7.75–7.76, indicating that these compounds were substituted at C-8 [23]; the chemical shift of H-5 was found as a singlet at *δ* 7.34–7.35. The furanoprotons appeared as doublets with* J *= 2.1–2.4 Hz. The resonance signals due to H-2′ and H-3′ were observed at *δ* 7.68-7.69 and 6.80–6.82, respectively. With respect to the compounds** 2**,** 5**–**8**, the resonance signals due to H-2′ and H-3′ were observed at *δ* 7.55–7.69 and 6.82–7.03, respectively. A downfield chemical shift of compounds** 2**,** 5**–**8**, for C-5, appeared at *δ* 147.9–151.2 indicating that these compounds were substituted at C-5, whereas compounds** 3 **and** 4 **contained resonance signals relatively upfield at *δ* 112.9–113.1 indicating that these compounds were substituted at C-8. Furthermore, compounds** 2**,** 5**–**8** were shown upfield of C-8 at *δ* 93.9–95.1 indicating that these compounds were unsubstituted at C-8. On the other hand, compounds** 3 **and** 4** were shown downfield of C-8 at *δ* 131.6–132.8 indicating that these compounds were substituted at C-8 [21]. Compound** 9** was identified as 3-*O*-glucopyranosyl-*β*-sitosterol from its mass spectrum and NMR spectroscopic measurements [24]. Inspection of the APT spectrum of compound** 9** showed the existence of 35 signals; the sugar moiety gives rise to one methylene group and five methine groups, whereas the aglycone part exhibits twenty-nine carbon signals, arising from six methyl groups, eleven methylene groups, nine methine groups, and three quaternary carbon atoms. The aglycone moiety was identified as *β*-sitosterol from its ^1^H and APT spectra. The sugar moiety was connected to C-3 atom as shown from its HMBC spectrum cross peaks H-1′/C-3.

All compounds are reported here for the first time in the* Ducrosia* genus except pabulenol.

### 4.2. Bioactivity Study

*D. enthifolia* is an herb used as additives in foods. Chemoprevention by dietary means continues to attract major attention for the management of chronic degenerative diseases primarily due to the dramatic rise of diabetes mellitus as major healthcare problem [3].

Cell damage has been implicated in a variety of chronic diseases. Oxidative stress and alterations in glucose metabolism are important risk factors for diabetes and its related complications. Advanced glycation end products (AGEs) and their carbonyl derivatives contribute to the pathogenesis of diabetes by their interaction with specific cell membrane receptors triggering to induce the expression of proinflammatory mediators and elicit oxidative stress, which exacerbate diabetic complications [3]. There is demonstrated evidence for the inactivation of glutathione synthesis in erythrocytes from type 2 diabetic patients and also a total reduction in antioxidant activity in both type 1 and type 2 diabetic patients [25].

One therapeutic approach for treating diabetes is to decrease the postprandial hyperglycemia by retarding the absorption of glucose through the inhibition of the carbohydrate hydrolyzing enzymes, *α*-amylase, *α*-glucosidase, and *β*-galactosidase, in the digestive tract. Inhibition of these enzymes delays carbohydrate digestion and prolongs overall carbohydrate digestion time, causing a reduction in the rate of glucose absorption and consequently blunting the postprandial plasma glucose rise [26].

Furanocoumarins provide examples of their versatility in a range of applications in the fields of biology and pharmacology. The administration of coumarin-rich fraction of* Ionidium suffruticosum* at the dose of 10 mg/kg BW/day along with high-fat diet significantly (*P* < 0.001) prevented the rise in the plasma total and LDL-c, triglycerides, and phospholipids and also showed cardioprotective effect against hyperlipidemia [27].

The isolated imperatorin (**4**) showed the highest activity against carbohydrate metabolizing enzyme (Table 1). In this context, Pari and Rajarajeswari [28] found that administration of coumarin to diabetic rats resulted in alterations in the metabolism of glucose with subsequent reduction in plasma glucose levels. Kleiner et al. [29] and Meijer and DePierre [30] reported the effect of imperatorin on hepatic drug-metabolizing enzymes of cytochromes P450 and glutathione S-transferases. It has diverse activity in terms of inducing various xenobiotic metabolizing enzymes. Woo and coworkers [31] investigated the effect of furanocoumarins isolated from* Angelica koreana *to retard the drug metabolism both* in vitro* and* in vivo*. These data agreed with the current* in vitro* study on carbohydrate metabolizing enzymes. In the present study, imperatorin (**4**) and 5-methoxypsoralen (**2**) showed the highest activity, while a moderate activity for psoralen (**1**), oxypeucedanin hydrate (**8**), and isooxypeucedanin (**5**) was recorded (Table 1).

A significant elevation in liver function markers associated with significant reduction in total protein content as compared to normal control group was illustrated (Table 2). The high serum levels of these enzymes after STZ treatment are associated with inflammation and/or injury to liver cells, a condition known as hepatocellular liver injury and apoptosis. In parallel with this a significant increased activitiy of serum enzymes relative to the normal level was previously revealed [31]. Supporting our findings, it has been found that hyperglycemia resulted in hepatolysis was reflected by increased blood serum aminotransferases as one of the consequences of diabetic complication. The increment of such serum markers may be due to the leakage of these enzymes from the liver cytosol into the blood stream as a result of hepatomegaly (fatty liver) [32].

The significant reduction in total protein content in diabetic rats is concomitant with the results of Otsuki and Williams [33], who found significant reduction in serum total protein concentrations in diabetic rats and this may be due to reduction in the three major phases in protein secretion, intracellular transport, and discharge. Alderson et al. [34] explained the reduction in total protein due to significant increase in protein excretion. Mendez et al. [35] reported that nonenzymatic glycation of albumin was the potential to alter its biological structure and function. It is mainly due to the formation of a Schiff base between amino group of lysine (and sometimes arginine) residues and excess glucose molecules in blood to form glycoalbumin. Hypoalbuminemia is one of the factors responsible for the onset of ascites related to liver fibrosis [36].

In the present study, a significant reduction in total cholesterol (TC) and triglycerides (TGs) after the treatment with* D. enthifolia* extract (Table 3) as compared to glibenclamide treated group [37, 38] was shown. These activities may be related to the presence of methoxypsoralens as 8-methoxypsoralen (**3**), which is in agreement with the reported data of Iyer and Patil [39].

The present results indicate significant elevation in NO and MDA levels in liver of diabetic rats (Table 4). These elevated levels may be due to oxidative stress which is considered one of the necessary causative factors that link diabetes with the pathogenic complications of several tissues [10]. Experimental studies suggested that NO may be responsible for the increased liver injury [40, 41].

Lipid peroxidation can damage protein, lipid, carbohydrates, and nucleic acids and is one of the risk factors of protein glycation. Moustafa [42] reported elevated rates of liver lipid peroxidation accompanied with deterioration in glucose tolerance in GSH-depleted rats. It has been suggested that in free radical initiating systems, the deterioration in glucose tolerance is attributed to impaired insulin action. Initiation of lipid peroxidation by free radicals, in the lipid moiety of the cell membrane, was supposed to result in distortion of the structural and functional integrity of the cell membrane or internal cellular components. This would interfere with the ability of insulin to initiate and propagate its normal sequence of actions which may account, at least in part, for STZ-induced hyperglycemia [42]. Moreover, the current data show also that STZ caused a reduction in GSH and SOD levels in the liver of diabetic rats (Table 4). The decrease in the activity of free radical scavenging enzyme SOD may be due to its inactivation caused by excess ROS production. SOD neutralizes superoxide as it cannot cross lipid membrane producing hydrogen peroxide. Hydrogen peroxide can cross biological membranes. Catalase detoxifies hydrogen peroxide which plays principle role in tissue damage. So the reduction in SOD may damage the first line of enzymatic defence against superoxide anion and hydrogen peroxide [41]. The significant depletion in GSH in liver of diabetic rats indicates damage to the second line of antioxidant defence [43]. This probably further exacerbates oxidative damage by adversely affecting critical GSH related processes such as free radical scavenging, detoxification of electrophilic compounds, modulation of cellular redox status and thiol-disulphide status of proteins, and regulation of cell signalling and repair pathways [44].

Concerning glucolytic (LDH, PK, and HK) and gluconeogenic (PEPCK) enzymes, a significant decrease in enzyme pathways was noticed in diabetic group as compared to normal control (Table 5). Sherlock and Dooley [45] found that, in diabetic state, degradation of liver glycogen and gluconeogensis may be increased and glycolysis is decreased, while glucose utilization is inhibited. Glucose-6-phosphatase increases in the liver, facilitating glucose release into the blood. The opposing enzyme which phosphorylates glucose, that is,hexokinase, is unaffected by insulin and decreases in diabetes. As a result, the liver continues to produce glucose even with severe hyperglycemia. It was found that, during renal dysfunction or renal damage, the concentration of the metabolites increased in blood that may be due to high activities of xanthine oxidase, lipid peroxidation, and increased triacylglycerol and cholesterol levels [10, 46]. Urea and creatinine are the main products of protein catabolism. The increase in serum urea and creatinine levels in STZ diabetic group indicates impairment in the normal kidney function of the animal, as the mechanism of removing them from the blood might have been affected. It may also be an indication of dysfunction at the glomerular and tubular levels of the kidney, and it is well known that many biochemical and histopathological findings confirmed renal damage in diabetic conditions [47].

In streptozotocin-induced hyperglycemic rats, glomerular alterations such as microalbuminuria, increase in urea levels, and decrease in creatinine clearance, as well as tubular disorders, were observed [41]. However, Zand Parsa et al. [48] found that the linear regression analysis revealed urinary albumin to creatinine ratio (UA/CR) as an independent predictor for the severity of coronary artery disease in DM type 2.

Oral administration of* D. anethifolia* total extract could reverse the above-mentioned diabetic effects. This may be through potentiating the pancreatic secretion of insulin from islet *β*-cells or the transport of blood glucose to the peripheral tissue. In addition* D. anethifolia* might increase the levels of insulin and* C*-peptide in diabetic [49].

One of the possible actions of* D. anethifolia* may be due to its inhibition of endogenous synthesis of lipids [46]. Furthermore, the crude* D. anethifolia* extract was found to have therapeutic potential due to its antioxidant activity in several areas, including the capacity of preventing and decreasing the damage caused by hyperlipidemia and hyperglycemia [50].

The enhanced hexokinase and other glucolytic enzyme activities (LDH, PK, and HK), with suppression in gluconeogenic enzyme PEPCK in plant extract treated rats (Table 5), suggested a greater uptake of glucose from blood by liver cells. The activities of enzymes suggested that enhanced lipid metabolism during diabetes is shifted towards carbohydrate metabolism and it enhances the utilization of glucose at peripheral sites.

In addition, the antihyperglycemic effect of different isolated furanocoumarin compounds could inform their stimulatory action on intracellular glucose transport by causing an increase in glucose uptake by different cells in absence of insulin and thus could attenuate cytokine-induced toxicity which reduce the oxidative damage of the pancreas and hence may ameliorate the endocrine function of this gland [51]. Also, stimulation of sirtuin SIRT1 (an enzyme which regulates the metabolic rate) enzyme activity decreases glucose levels and improves insulin sensitivity. Imperatorin (**4**) was reported to be an active constituent increasing* in vitro* insulin release to 170.3% [52].

The crude extract of* D. anethifolia* showed a decrease in the blood glucose in rats with hyperglycemia when compared to the standard antidiabetic drug glibenclamide [53]. These results may be attributed to its antidiabetic effect, which is mainly due to the content of bioactive furanocoumarins as imperatorin, methoxypsoralens, and the other linear furanocoumarins [54].

This research suggests that* D. anethifolia* offers an attractive potential strategy to regulate postprandial hyperglycemia toward an overall dietary support for type 2 diabetes management.

## 5. Conclusion

It could be concluded that the crude extract and its major isolated furanocoumarins exhibited* in vitro* inhibitory effects against carbohydrate metabolizing enzymes (*α*-amylase, *α*-glucosidase, and *β*-galactosidase) in a concentration dependent relationships. Imperatorin and 5-methoxypsoralen showed the most potent inhibitory power, while psoralen, oxypeucedanin hydrate, and isooxypeucedanin showed moderate inhibitory activities. The biological activity of* in vivo D. anethifolia *crude extract showed hypoglycemic, hypolipidemic, and antioxidant effects as well as an amelioration in kidney function through improvement in total urea and creatinine levels.

## Figures and Tables

**Table 1:** * In vitro* carbohydrate metabolizing enzymes inhibition activities (%) of* Ducrosia anethifolia* total extract and isolated linear furanocoumarins.

Concentrations (*µ*g/mL)	Acarbose (positive control)	Total extract	Psoralen (**1**)	5-methoxypsoralen (**2**)	Imperatorin (**4**)	Isooxypeucedanin (**5**)	Oxypeucedanin hydrate (**8**)
			*α*-amylase inhibition%				
10	32.20 ± 1.29^a^	31.20 ± 2.34^a^	16.48 ± 0.98^e^	17.59 ± 0.60^e^	28.27 ± 2.97^d^	19.21 ± 1.08^e^	18.40 ± 1.10^e^
50	35.19 ± 348^c^	29.33 ± 4.13^a^	24.78 ± 2.24^b^	28.70 ± 1.04^a^	34.16 ± 1.04^c^	22.11 ± 1.22^b^	23.33 ± 2.45^b^
100	47.37 ± 4.15^g^	45.00 ± 5.06^g^	28.34 ± 1.89^e^	34.29 ± 3.11^d^	45.15 ± 6.18^g^	24.14 ± 2.00^f^	25.30 ± 1.99^f^
500	52.55 ± 4.49^c^	49.97 ± 6.19^c^	39.65 ± 2.80^b^	45.12 ± 3.33^a^	52.26 ± 5.69^c^	39.77 ± 3.20^b^	37.08 ± 2.00^b^
1000	71.34 ± 2.65^d^	70.77 ± 8.87^d^	50.50 ± 1.10^b^	58.10 ± 2.14^a^	67.56 ± 4.67^c^	51.20 ± 3.40^b^	53.50 ± 1.10^b^
LSD 5%	5.88	4.99	3.9	4.32	5.18	4.99	7.90

			*α*-glucosidase inhibition%				
10	29.94 ± 2.04^b^	28.89 ± 6.67^b^	23.00 ± 3.03^c^	25.15 ± 3.08^c^	28.89 ± 2.90^b^	24.33 ± 2.00^c^	24.28 ± 2.13^c^
50	43.25 ± 3.09^c^	40.20 ± 6.79^c^	33.55 ± 4.73^b^	34.81 ± 23.68^b^	37.76 ± 6.18^a^	32.12 ± 1.28^b^	31.68 ± 5.00^b^
100	52.45 ± 4.67^d^	47.30 ± 5.58^c^	41.65 ± 2.00^a^	45.20 ± 2.45^d^	46.56 ± 2.46^c^	39.90 ± 6.50^a^	40.23 ± 4.56^a^
500	69.14 ± 4.15^c^	56.25 ± 7.29^b^	42.75 ± 1.79^a^	48.00 ± 2.78^d^	55.67 ± 6.78^b^	41.51 ± 2.88^a^	42.91 ± 2.60^a^
1000	85.33 ± 2.47^c^	74.25 ± 8.09^b^	59.85 ± 2.90^e^	64.39 ± 1.78^d^	69.66 ± 3.67^b^	52.31 ± 2.50^a^	51.67 ± 2.60^a^
LSD 5%	5.90	10.99	4.90	5.93	5. 94	5.77	5.99

			%				
10	45.85 ± 2.90^a^	42.20 ± 5.57^a^	17.90 ± 4.32^c^	23.56 ± 4.31^b^	34.54 ± 4.20^d^	19.90 ± 6.22^c^	18.90 ± 2.32^c^
50	55.90 ± 4.70^a^	50.00 ± 8.90^d^	35.35 ± 9.00^b^	34.77 ± 5.90^b^	39.79 ± 5.90^c^	33.90 ± 4.40^b^	33.26 ± 5.06^b^
100	59.00 ± 6.10^a^	55.67 ± 8.80^h^	37.86 ± 5.48^c^	39.46 ± 6.35^b^	53.90 ± 6.80^d^	36.30 ± 4.35^c^	35.80 ± 5.67^c^
500	78.56 ± 5.78^a^	77.30 ± 4.56^a^	54.33 ± 5.41^c^	62.34 ± 5.99^b^	69.90 ± 6.50^d^	50.70 ± 4.71^c^	48.70 ± 3.56^e^
1000	83.66 ± 6.51^a^	78.66 ± 9.01^h^	56.00 ± 5.01^c^	65.31 ± 4.17^b^	72.86 ± 4.41^d^	54.34 ± 4.57^c^	56.09 ± 4.41^c^
LSD 5%	11.00	11.00	13.1	12.7	11.20	12.99	12.96

Data are means ± SD of three replicates in each group. Statistical analysis is carried out using CoStat Computer Program coupled with post hoc (least significance difference LSD), where the unshared letter is significantly different at *P* ≤ 0.05.

**Table 2:** Effect of *Ducrosia anethifolia* total extract on blood glucose level and liver injury biomarkers in STZ-induced diabetes in rats.

Parameter	Normal control	*D. anethifolia* treated normal	STZ treated	STZ +* D. anethifolia* treated	STZ + glibenclamide
Blood glucose level	111.53 ± 3.33^a^	116.58 ± 4.00^a^	365.00 ± 23.20^b^	165.60 ± 8.30^d^	151.50 ± 2.10^c^
AST	2.55 ± 0.19^a^	2.43 ± 0.20^a^	8.15 ± 0.18^b^	3.20 ± 0.21^a^	2.85 ± 0.05^a^
ALT	1.62 ± 0.028^a^	1.52 ± 0.09^a^	5.92 ± 0.17^b^	2.10 ± 0.23^c^	2.00 ± 0.16^c^
ALP	3.46 ± 0.08^a^	3.61 ± 0.06^a^	10.90 ± 0.19^b^	5.00 ± 0.13^a^	4.03 ± 0.38^a^
Total protein	117.47 ± 16.00^a^	119.90 ± 9.22^a^	65.48 ± 6.49^b^	100.70 ± 9.21^a^	107.00 ± 2.64^a^

Data are means ± SD of ten rats in each group. Liver injury biomarkers activities are expressed in *μ*mol/mL (unit/mL).

Total protein is expressed in mg/mL. Statistical analysis is carried out using CoStat Computer Program coupled with post hoc (least significance difference LSD. The different groups were compared with each other at the same time and the unshared letter is significantly different at *P* ≤ 0.05.

**Table 3:** Effect of *Ducrosia anethifolia* total extract on lipid profile in STZ-induced diabetes in rats.

Groups	Parameters				
	Normal control	*D. anethifolia* treated normal	STZ treated	STZ +* D. anethifolia* treated	STZ + glibenclamide
TC	49.60 ± 10.95^a^	53.03 ± 5.45^a^	160.00 ± 15.00^b^	87.00 ± 9.23^d^	84.69 ± 8.24^d^
TG	24.72 ± 7.16^a^	25.20 ± 2.00^a^	67.00 ± 10.00^b^	39.40 ± 1.90^c^	38.18 ± 1.57^c^
HDL	30.21 ± 2.48^a^	31.29 ± 3.16^a^	9.15 ± 0.97^b^	18.23 ± 1.09^c^	18.82 ± 1.62^c^
LDL	12.49 ± 9.24^a^	13.00 ± 1.09^a^	98.10 ± 14.56^b^	39.96 ± 11.00^c^	38.23 ± 4.65^d^
Total lipids	1100.00 ± 52.6^a^	1105.26 ± 52.65^a^	2150.9 ± 99.50^b^	1510.66 ± 60.20^c^	1421.00 ± 59.60^c^

Data are means ± SD of ten rats in each group. Lipid profile parameters are expressed in ug/dL except HDL which is expressed in mg/dL. Statistical analysis is carried out using CoStat Computer Program coupled with post hoc (least significance difference LSD). Unshared letters indicate significant correlation at *P* < 0.05.

**Table 4:** Effect of *Ducrosia anethifolia* total extract on NO, MDA levels, antioxidant, and antioxidant enzyme in STZ-induced diabetes in rats.

Groups	Parameters				
	Normal control	*D. anethifolia* treated normal	STZ treated	STZ +* D. anethifolia* treated	STZ + glibenclamide
NO	43.68 ± 0.7^a^	43.50 ± 4.13^a^	78.19 ± 1.00^b^	51.05 ± 3.21^c^	51.00 ± 9.91^c^
MDA	18.53 ± 1.19^a^	17.56 ± 0.29^a^	124.11 ± 8.47^b^	25.36 ± 4.53^c^	23.10 ± 4.63^c^
GSH	3.40 ± 0.30^a^	3.61 ± 0.34^a^	1.01 ± 0.05^b^	2.45 ± 0.23^c^	2.67 ± 0.38^c^
SOD	9.88 ± 0.67^a^	8.78 ± 0.18^a^	1.67 ± 0.17^b^	6.00 ± 1.10^c^	7.00 ± 1.30^c^

Data are means ± SD of ten rats in each group. Blood glucose level is expressed in mg/dL. MDA is expressed in *μ*moL/min/g tissue. GSH is expressed as *μ*g glutathione/mg protein and SOD is expressed in *μ*mol/mg protein/min. NO is expressed in *μ*g/g tissue. Statistical analysis is carried out using one-way analysis of variance (ANOVA) using CoStat Computer Program. The different groups were compared with each other at the same time and the unshared letter is significantly different at *P* ≤ 0.05.

**Table 5:** Effect of *Ducrosia anethifolia* total extract on some glucolytic and gluconeogenic enzymes in STZ-induced diabetes in rats.

Groups	Parameters				
	Normal control	*D. anethifolia* treated normal	STZ treated	STZ +* D. anethifolia* treated	STZ + Glibenclamide
HK	105.12 ± 1.67^a^	102.00 ± 9.00^a^	35.19 ± 7.60^b^	57.18 ± 0.95^c^	76.99 ± 8.89^d^
PK	62.60 ± 2.45^a^	64.69 ± 3.41^a^	23.40 ± 5.17^b^	47.47 ± 2.03^c^	50.16 ± 9.05^d^
LDH	39.27 ± 9.80^a^	39.00 ± 5.52^a^	18.41 ± 2.62^b^	29.90 ± 5.90^c^	31.40 ± 8.48^d^
PEPCK	3.40 ± 0.30^a^	3.76 ± 0.45^a^	1.16 ± 0.20^b^	2.58 ± 0.23^c^	2.87 ± 0.28^d^

Data are means ± SD of ten rats in each group. Blood glucose level is expressed in mg/dL. PK, HK, LDH and PEPCK are expressed in *μ*mol/mg protein/min. Statistical analysis is carried out using Co stat Computer Program coupled with post-hoc (least significance difference LSD. The different groups were compared with each other at the same times and the unshared letter is significant difference at *P* ≤ 0.05.

**Table 6:** Effect of *Ducrosia anethifolia* total extract on total urea and creatinine levels in STZ-induced diabetes in rats.

Groups	Parameters				
	Normal control	*D. anethifolia* treated normal	STZ treated	STZ +* D. anethifolia* treated	STZ + glibenclamide
Total urea	36.00 ± 6.10^a^	34.20 ± 3.80^a^	89.00 ± 2.80^b^	46.45 ± 3.07^c^	33.23 ± 6.90^a^
Creatinine	0.84 ± 0.04^a^	0.80 ± 0.05^a^	3.90 ± 0.20^b^	1.09 ± 0.12^c^	0.80 ± 0.04^a^

Data are means ± SD of ten rats in each group. Total urea and creatinine are expressed in mg/dL.

Statistical analysis is carried out using CoStat Computer Program coupled with post hoc (least significance difference LSD). The different groups were compared with each other at the same time and the unshared letter is significantly different at *P* ≤ 0.05.
